# Evidence of a sudden increase in the nuclear size of proton-rich silver-96

**DOI:** 10.1038/s41467-021-24888-x

**Published:** 2021-07-28

**Authors:** M. Reponen, R. P. de Groote, L. Al Ayoubi, O. Beliuskina, M. L. Bissell, P. Campbell, L. Cañete, B. Cheal, K. Chrysalidis, C. Delafosse, A. de Roubin, C. S. Devlin, T. Eronen, R. F. Garcia Ruiz, S. Geldhof, W. Gins, M. Hukkanen, P. Imgram, A. Kankainen, M. Kortelainen, Á. Koszorús, S. Kujanpää, R. Mathieson, D. A. Nesterenko, I. Pohjalainen, M. Vilén, A. Zadvornaya, I. D. Moore

**Affiliations:** 1 University of Jyväskylä, Jyväskylä, Finland; 2 CNRS/IN2P3, IJCLab, Université Paris-Saclay, Orsay, France; 3 School of Physics and Astronomy, University of Manchester, Manchester, UK; 4 University of Surrey, Guildford, UK; 5 Department of Physics, University of Liverpool, Liverpool, UK; 6 CERN, Geneva, Switzerland; 7 CNRS/IN2P3 Université de Bordeaux, Centre d’Etudes Nucléaires de Bordeaux Gradignan, Gradignan Cedex, France; 8 Massachusetts Institute of Technology, Cambridge, MA USA; 9 Instituut voor Kern-en Stralingsfysica, KU Leuven, Leuven, Belgium; 10 Technische Universität Darmstadt, Department of Physics, Institut für Kernphysik, Darmstadt, Germany; 11 GSI Helmholtzzentrum für Schwerionenforschung GmbH, Darmstadt, Germany

**Keywords:** Experimental nuclear physics, Theoretical nuclear physics

## Abstract

Understanding the evolution of the nuclear charge radius is one of the long-standing challenges for nuclear theory. Recently, density functional theory calculations utilizing Fayans functionals have successfully reproduced the charge radii of a variety of exotic isotopes. However, difficulties in the isotope production have hindered testing these models in the immediate region of the nuclear chart below the heaviest self-conjugate doubly-magic nucleus ^100^Sn, where the near-equal number of protons (*Z*) and neutrons (*N*) lead to enhanced neutron-proton pairing. Here, we present an optical excursion into this region by crossing the *N* = 50 magic neutron number in the silver isotopic chain with the measurement of the charge radius of ^96^Ag (*N* = 49). The results provide a challenge for nuclear theory: calculations are unable to reproduce the pronounced discontinuity in the charge radii as one moves below *N* = 50. The technical advancements in this work open the *N* = *Z* region below ^100^Sn for further optical studies, which will lead to more comprehensive input for nuclear theory development.

## Abstract (web summary)

Laser spectroscopic measurements of isotopes near the doubly-magic 100-Sn are challenging due to difficulties in their production. Here the authors measure the ground state charge radius of the proton-rich 96-Ag isotope and find a discontinuity in the nuclear size when crossing the neutron number N equal to 50.

## Introduction

The region of the nuclear chart below the heaviest self-conjugate doubly-magic nucleus, ^100^Sn, is a continuing subject for intense theoretical and experimental studies. The closely equal number of protons and neutrons (*N* = *Z*) in the regions atomic nuclei leads to enhanced neutron-proton pairing^1,2^, thus posing a fertile ground for testing the validity of shell-model (SM) predictions and for improving our understanding of the proton–neutron (p–n) interaction. The region is also rich in isomers^3–6^, some of which exhibit unique features^7^, and the astrophysical rapid-proton capture process (rp-process), powering type I x-ray bursts^8,9^, traverses through these nuclei. Despite the interest, experimental data on the ground-state properties of these nuclei are scarce^10^.

Laser spectroscopy is an efficient method for the determination of nuclear ground- and isomeric-state properties. Measurements of atomic hyperfine structure and isotope shifts provide nuclear-model independent access to the nuclear spin, magnetic dipole, and electric quadrupole moments as well as changes in root-mean-square charge radii^11^. The nuclear charge radius provides insight into the shell and subshell effects^12^, correlations and mixing^13^, and nuclear deformation^14^. The recent demonstration of the charge radii’s sensitivity to aspects of nuclear structure^15^ has allowed testing of ab initio methods, density functional approaches, and SM calculations^16^.

In this article, we present a laser spectroscopic excursion near ^100^Sn and below *N* = 50, with measurements of the mean-squared charge radii of ^96−104^Ag with in-source resonance ionization spectroscopy (RIS) complemented by collinear laser spectroscopy of ^114−121^Ag. The study of ^96^Ag (*Z* = 47, *N* = 49) was made possible with a tailor-made method, combining efficient in-source RIS in a hot-cavity catcher with sensitive ion detection via the phase-imaging ion-cyclotron resonance (PI-ICR) Penning trap mass spectrometry^17^ technique. The importance of these results is demonstrated through comparisons to density functional theory (DFT) calculations with a Fayans functional coupled to a deformed implementation.

## Results

### Experimental setup

**Fig. 1:**
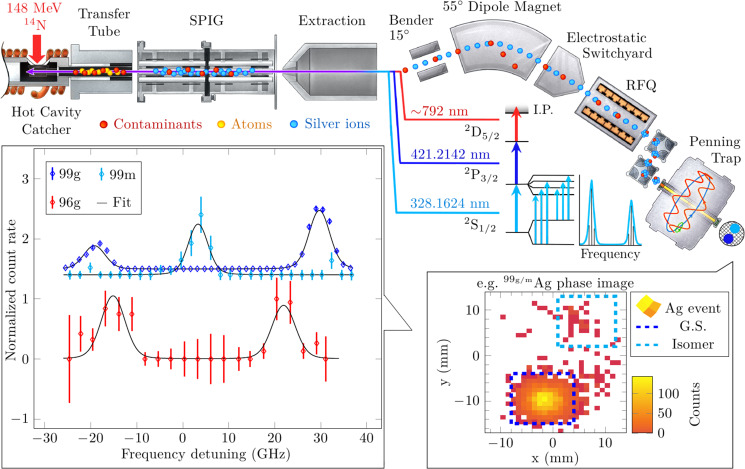
Overview of the experimental method. A 148 MeV ^14^N beam from the K-130 cyclotron impinges on a ~ 3 mg cm^−2^ ^92/nat.^Mo target and produces silver nuclei in the region of *N* = 50 via fusion–evaporation reactions. The products recoil out of the target, implant into hot graphite, and promptly diffuse out to the catcher cavity before effusing into a transfer tube. The atoms overlap with counter-propagating laser beams and undergo a three-step resonance laser ionization process. The silver ions, along with surface ions and sputtered target-like contaminants, are then guided into the sextupole ion guide (SPIG) by a ~ 1 V per cm field gradient along the tube, and subsequently accelerated to 30 keV, mass separated, and introduced into the radiofrequency quadrupole (RFQ) cooler-buncher. The ions are released as bunches and injected into the JYFLTRAP Penning trap for mass analysis and detection via the PI-ICR method. At the last stage, software processing of the laser frequency-tagged ion impact location data yields the hyperfine spectrum, presented here for ^99^Ag ground-state and isomer (99g and 99m, respectively) and for the ^96^Ag ground-state (96g). The error bars indicate the statistical error.

The experiments took place at the Ion Guide Isotope Separation On-Line (IGISOL) facility at the University of Jyväskylä Accelerator Laboratory^18^. Our approach is three-fold, realized with an experimental setup presented in Fig. 1. Firstly, for silver nuclei with *N* > 51, the ^92^Mo(^14^N, 2pxn)^104−99^Ag heavy-ion fusion–evaporation reaction offered the optimal production yields with respect to available primary beam intensity, as in a previous study^19^. While the cross-sections for producing ^96−98^Ag were lower compared to other possible reactions^3,19^, the advancements in the production and detection techniques, described below, enabled the probing of even these highly challenging cases with the same reaction.

Secondly, we implemented a hot-cavity catcher laser ion source^20,21^ for fast, high-efficiency stopping, and extraction of these reaction products^22,23^. The produced silver nuclei, which recoil out of the target, implant into a hot graphite catcher from where they promptly diffuse out into the catcher cavity as atoms, before effusing into a transfer tube. There, the silver atoms are selectively ionized with a three-step laser ionization scheme. Compared to the standard gas-cell approach in use at the IGISOL facility, this method has the advantage of a very high stopping efficiency while neutralizing the reaction products for a subsequent selective re-ionization.

Lastly, we utilize the well-established coupling of RIS to mass spectrometry. The ions are mass separated in the JYFLTRAP double Penning trap^24^, using the PI-ICR method^17^, and tagged with the frequency of the first resonant step in the three-step scheme. The high mass resolving power offered by the PI-ICR method enables simultaneous measurement of the hyperfine structure of multiple long-lived, (*t*_1/2_ > 100 ms), nuclear states with mass differences as low as ~10 keV, in an essentially background-free manner. However, even though long-lived low-spin isomers have been observed in nearby silver isotopes^3,25,26^, the cross-section for producing these states with our reaction is orders of magnitude smaller than for the higher-spin states. Thus, an isomeric state was only observed for the high-yield case of ^99^Ag (*E*_ex_ = 506.2 keV, *I* = (1/2^−^)) within the time constraints of the measurement. The RIS of ^96^Ag, with on-resonance signal rates as low as ~0.005 ions per second, demonstrates the immediate potential of this PI-ICR assisted in-source RIS technique. These rates correspond to an average cross-section of the order of one *μ*barn, estimated using the PotFus^27^ and GEMINI++ fusion–evaporation codes^28^ as presented in ref. ^29^. Measurements with such low event rates are only possible if the background rate is similarly low, a condition which is satisfied with the PI-ICR method.

### Laser spectroscopy of silver

A step size of ≈300 MHz was used for scanning the laser frequency over the hyperfine structure of the proton-rich silver isotopes resulting in multiple data points within the resonance full-width half maximum (FWHM) of about 5 GHz. The FWHM is composed of the laser linewidth of about ~3 GHz in the third-harmonic, with an additional contribution of ~3 GHz due to the Doppler broadening caused by the hot-cavity temperature of about 1800 K. The laser power was not optimized to minimize the power broadening component due to a lack of power stabilization. This linewidth is sufficiently narrow to extract reliable magnetic dipole moments and the changes in mean-squared charge radii. Several sweeps across the entire frequency range were performed for every hyperfine scan, accumulating about 10 s of measurement time per data point, per sweep, with the total measurement time varying from a few tens of minutes to 12 h in the case of ^96^Ag. The methods section describes how the hyperfine spectra are constructed from the PI-ICR data and outlines the details of the data analysis. From the analysis, the isotope shift *δν*^109,*A*^ = *ν*^*A*^ − *ν*^109^ between the centroid of the isotope of interest *ν*^*A*^ and the reference isotope ^109^Ag, *ν*^109^, was extracted. The magnetic moments determined for ^97−104^Ag in the PI-ICR assisted in-source RIS agree well with the literature values, and we report ${\mu }_{exp}\,=\,4.5{7}_{-0.18}^{+0.28}{\mu }_{N}$ for ^96^Ag${}^{({8}^{+})}$. A detailed discussion of the magnetic moment is, however, beyond the scope of this work.

We complemented these measurements with high-precision studies using conventional collinear laser spectroscopy. This technique, used to probe neutron-rich ^114−121^Ag produced in proton-induced fission of uranium, is feasible for silver isotopes with production rates as low as 1000 ions per second, and features linewidths of about 75 MHz. The nuclear properties were inferred from the same 328 nm optical transition as the in-source measurements. We restrict the high-precision dataset of these neutron-rich isotopes to the nuclear ground-states; data obtained on the isomeric states are still undergoing analysis. By extending the measurements performed from below *N* = 50 into the mid-shell region, and towards *N* = 82, more stringent tests of the capabilities of the theoretical models are possible. Furthermore, with data spanning 19 silver isotopes, atomic mass- and field-shift constants required to extract nuclear charge radii from optical data can be validated. More details on the production, ion beam manipulation, measurement and analysis protocol of collinear laser spectroscopy can be found in the Methods section. Table 1 presents the measured isotope shifts compared to literature values, where available.

**Table 1:** Isotope shifts and the change in the mean-squared charge radii of Ag isotopes.

*A*	*δν* (GHz)	$\delta {\nu }_{\,{{\mbox{lit}}}\,}^{a}$ (GHz)	*δ*〈*r*^2^〉 (fm^2^)
96^a^	$2.6{4}_{-0.78}^{+1.12}$		$-1.2{1}_{-0.19}^{+0.27}\,[14]$
97	$4.2{9}_{-0.30}^{+0.33}$	4.67 (66) [8]	$-1.5{5}_{-0.08}^{+0.08}\,[15]$
98	$3.6{8}_{-0.23}^{+0.25}$	3.58 (47) [8]	$-1.3{6}_{-0.06}^{+0.06}\,[13]$
99	$3.2{0}_{-0.09}^{+0.10}$	3.21 (25) [11]	$-1.2{0}_{-0.02}^{+0.03}\,[12]$
99m	$3.1{3}_{-0.91}^{+1.07}$	4.00 (94) [11]	$-1.1{8}_{-0.22}^{+0.26}\,[12]$
100	$2.6{6}_{-0.33}^{+0.33}$	2.95 (22) [11]	$-1.0{2}_{-0.08}^{+0.08}\,[11]$
101	$2.6{8}_{-0.41}^{+0.42}$	2.48 (26) [11]	$-0.9{8}_{-0.10}^{+0.10}\,[10]$
102^a^	$1.6{0}_{-0.17}^{+0.17}$		$-0.6{7}_{-0.04}^{+0.05}\,[8]$
103	$1.5{8}_{-0.30}^{+0.30}$		$-0.6{3}_{-0.07}^{+0.07}\,[7]$
104	$1.7{1}_{-0.22}^{+0.22}$		$-0.6{1}_{-0.06}^{+0.06}\,[6]$
107^b^	0.467 (4)		−0.198 (2) [20]
109	0.0		0.0
114^a, b^	−0.850 (3)		0.384 (1) [50]
115^a, b^	−0.995 (5)		0.454 (3) [60]
116^a, b^	−1.040 (9)		0.500 (10) [60]
117^a, b^	−1.181 (6)		0.568 (3) [70]
118^a, b^	−1.203 (5)		0.607 (3) [80]
119^a, b^	−1.348 (5)		0.675 (3) [90]
120^a, b^	−1.379 (4)		0.715 (2) [90]
121^a, b^	−1.461 (3)		0.767 (1) [100]

**Fig. 2:**
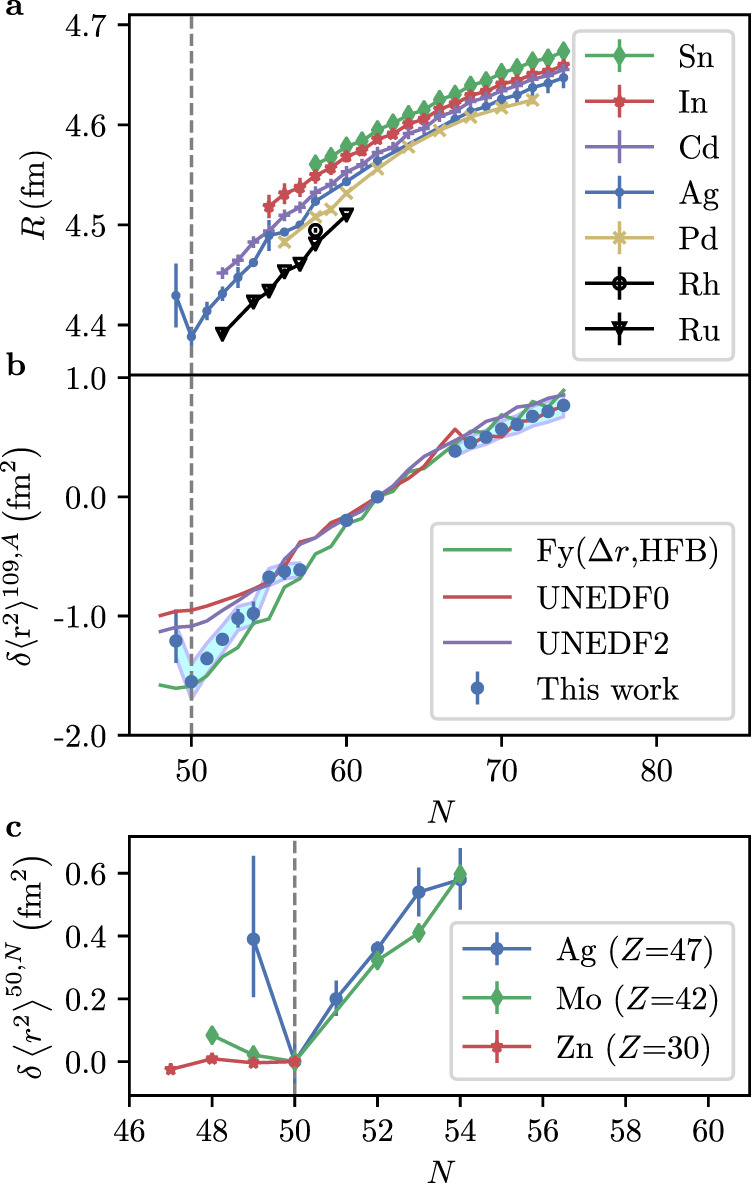
Comparison of experimental Ag data to theoretical calculations and other isotopic chains. **a** Experimental charge radii of Sn, In, Cd, Ag, Pd, Rh, and Ru isotopes up to *N* = 74^33–35^. The obtained empirical atomic parameters for the 328 nm transition aided in the recalculation of the charge radii of Ag in cases where literature isotope shifts were available. Similar treatment yielded empirical atomic parameters for the 547.7 nm transition^30^. Where multiple values were available, a weighted mean with the uncertainty given by the standard error of the weighted mean is shown. For asymmetric errors, the larger one is used. **b** Ground-state change in mean-squared charge radii for ^96−104^Ag (in-source RIS) and ^114−121^Ag (collinear laser spectroscopy). The data are compared to theoretical calculations with Fy(Δ*r*, Hartree-Fock-Bogoliubov (HFB))^43^, UNEDF0^41^ and UNEDF2^42^ EDFs. The error bars indicate the statistical error. The systematic error, due to the uncertainty in the atomic parameters, is indicated by the shaded band. **c** The change in the charge radii for Zn^38^, Mo^39^, and Ag near *N* = 50 illustrate an increasing trend in the magnitude of the kink as a function of proton number towards ^100^Sn. The error bars indicate the statistical error.

The isotope shifts (*δν*) for the 328.1624 nm, [Kr]4d^10^5s^2^S_1/2_ → 5p^2^P_3/2_ transition and corresponding charge radii from this work compared to literature isotope shift values (see Fig. 2b). The statistical error is given in super- and subscripts or in parentheses, and the systematic error is presented in square brackets. The reference isotope shift data *a* is from ref. ^19^.

^a^The isotopes with charge radii data only from this work.

^b^Collinear laser spectroscopy measurements.

The change in the mean-squared charge radii $\delta {\langle {r}^{2}\rangle }^{109,A}$ is related to the isotope shift as

1 $$\delta {\nu }^{109,A}\,=\,M\frac{{m}_{A}\,-\,{m}_{109}}{{m}_{A}\,\times\, {m}_{109}}\,+\,F\,\, K\delta {\langle {r}^{2}\rangle }^{109,A}$$

where *M* and *F* are the atomic mass and field-shift constants, respectively, and *m*_*A*,109_ are the atomic masses for the isotope of interest and ^109^Ag. The factor *K* corrects for higher order radial moments, differing from unity by only a few percent even for very heavy nuclei. In this mass region, *K* = 0.976^30^. Accurate values for the atomic factors *M* and *F* are not available from experimental data alone since there are only two stable silver isotopes and thus a King plot procedure is not possible; instead, atomic structure calculations are, in principle, required^31^. Employing the values of *M* and *F* used in previous work^19,30^ yields a charge radius trend that does not agree well with those observed in neighboring isotope chains. We therefore performed an empirical calibration by retaining the previously used value^19^ of *F* = −4300 (300) MHz fm^−2^, and adjusting the value of *M* to match the charge radius difference of the two stable silver isotopes to the literature difference^32^ of *δR*_109,107_ = 0.021 fm. This procedure yields *M* = 1956 (360) GHz u; the uncertainty is due to the propagation of the uncertainty in *F*. Literature data using the 547.7 nm transition from ref. ^30^ was empirically calibrated in a similar fashion for consistency. The comparison with other isotopic chains^33–35^, shown in Fig. 2a, which was made possible due to the additional data points on the neutron-rich side obtained with collinear laser spectroscopy, validates this empirical calibration.

## Discussion

This work reveals a kink in the charge radius when crossing *N* = 50, as presented in Fig. 2, despite the rather large uncertainty on the radius of ^96^Ag. Such discontinuities have often been observed at the crossing of magic numbers, for example at *N* = 28^16^ and *N* = 82^36^, providing support for the role of magicity at *N* = 50 in the vicinity of ^100^Sn. Recently, magicity was also demonstrated on the far neutron-rich side of stability^37^ in ^78^Ni (*N* = 50). As our data represent the only crossing of *N* = 50 in the immediate vicinity of ^100^Sn, it is instructive to compare the $\delta {\left\langle {r}^{2}\right\rangle }^{N \,=\, 49,\,50}$ values of silver with other isotope chains to investigate the relative magnitude of the kink in the charge radius at *N* = 50, as shown in Fig. 2c. At zinc^38^ (*Z* = 30), the lightest isotone with a measured charge radius at *N* = 50, the $\delta {\left\langle {r}^{2}\right\rangle }^{49,50}$ is essentially zero. In the molybdenum chain (*Z* = 42), the nearest element to silver with experimental data at *N* = 50^39^, a minor increase in the radius is observed for *N* = 49 relative to *N* = 50. With five additional protons compared to molybdenum, silver exhibits a larger increase in charge radius, perhaps indicating an increasing trend in magnitude towards doubly-magic ^100^Sn.

In order to investigate these qualitative observations in more detail, DFT calculations, using Fayans (Fy)^40^ and UNEDF^41,42^ Skyrme Energy Density Functionals (EDF), were performed. Recently, the effectiveness of the Fy(Δ*r*, HFB)^43^ parameterization on reproducing total radii^14,35,36,44^ as well as smaller odd-even variations, such as the odd-even staggering in copper^13^ isotopes, was demonstrated. The (Δ*r*) parametrization has furthermore demonstrated the ability to reproduce the changes of nuclear charge radii when crossing shell closures for both proton-rich calcium (*Z* = 20) isotopes^44^ as well as neutron-rich Sn (*Z* = 50)^36^ isotopes. These successes make the Fayans functional a prime candidate to investigate the trend of the silver radii. We note, in this work, the results with Fayans functionals were calculated with adjusted pairing strength due to differences between DFT solvers used in original parameter adjustment (coordinate space solver) and present calculations (harmonic oscillator basis).

Figure 2b compares all models with the experimental changes in mean-squared charge radii. In general, the UNEDF EDFs predict a rather smooth behavior, while the Fayans EDF more closely follows the local variation in charge radii. All functionals provide a very good reproduction of the experimental trend for the neutron-rich isotopes. The odd-even staggering obtained with Fy(Δ*r*, HFB) is however too large, a phenomenon which was also observed to a similar extent in the potassium^45^ (*Z* = 19), copper^13^ (*Z* = 29), and cadmium^35^ (*Z* = 48) isotope chains. More striking is the deviation of all models near *N* = 50: none of the models can reproduce the pronounced increase of the measured radius of ^96^Ag below *N* = 50. The notable difference between UNEDF and Fy(Δ*r*, HFB) functionals in the vicinity of the shell closure, also present in calculated absolute values, may arise from the differences in the infinite nuclear matter properties between the models. The nuclear matter saturation density is higher with Fy(Δ*r*, HFB) than with the UNEDF models, resulting therefore in a smaller nucleus.

It is very unlikely that the increase of the charge radius seen in ^96^Ag can be reproduced with any reasonable EDF model which is based on a deformed mean-field. In this approach, the mean-field and resulting wavefunction breaks, for example, rotational symmetry, which would be conserved by the underlying nucleon-nucleon interaction. The wavefunction is no longer an eigenstate of angular momentum. A possible solution towards a more accurate description of the charge radius could be through the restoration of broken symmetries. The symmetry-restored wavefunction becomes a linear combination of multiple symmetry-broken wavefunctions, incorporating correlations beyond the mean-field. This has the potential to better describe the local variation of the charge radius.

**Fig. 3:**
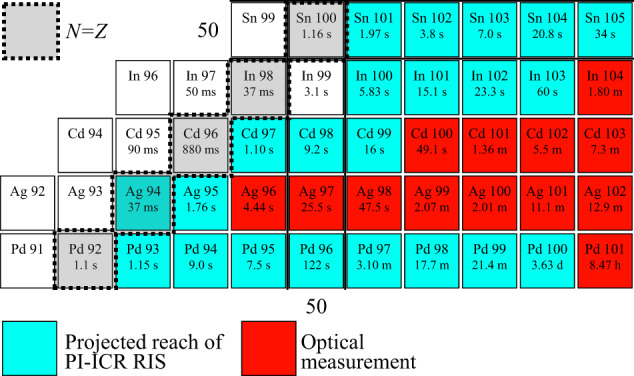
Outlook for the PI-ICR -assisted RIS. The figure presents the status of optical measurements in the ^100^Sn region of the nuclear chart and the projected reach of the PI-ICR-assisted RIS technique at IGISOL. The projections are based on LISE++ simulations and Gemini++ cross-section calculations. They assume a 0.5% efficiency after mass separation and a 10% efficiency through the RFQ and Penning trap as seen in Fig. 1. The absolute laser ionization efficiency for these elements ranges from below 9% in Sn^51^ to above 50% in Pd^52^. Only for In the absolute value is not known, however in this estimate we assume it to be of the order of 10%. Depending on the isotope of interest, the primary beam is either ^40^Ca or ^58^Ni, with a projected conservative 50 pnA intensity. With these assumptions, similar statistics as for ^96^Ag can be collected for even the most exotic cases in <12 h of laser spectroscopy. Furthermore, each of the most exotic cases per element have already been extracted from a hot cavity^7,21,48–50^.

Beyond silver, our combination of techniques can be used to study other elements in the *N* = *Z* region near ^100^Sn. Cross-section calculations, validated by experiments at the GSI Helmholtzzentrum für Schwerionenforschung^7,21,46–50^, together with high laser ionization efficiencies^51,52^ and our primary beam intensities suggest ^93^Pd, ^97^Cd, ^100^In, and ^101^Sn are within reach, as highlighted in Fig. 3.

Laser spectroscopy as well as precise mass measurements can be exploited in parallel, helping to elucidate the structural evolution of nuclei along the *N* = *Z* line. Phenomena arising from enhanced correlations between neutrons and protons occupying orbitals with identical quantum numbers remains a challenge to understand. For example, a clear fingerprint for spin-aligned neutron-proton pairing remains an open question^53^, the evidence for this effect in *N* = *Z* nuclei^2,54^ calling for further probing of charge radii of self-conjugate nuclei in the region. The recently demonstrated treatment of p–n pairing in a symmetry-restored mean-field approach^55^ has opened a path for the development of DFT models to elucidate this phenomenon. Furthermore, while the charge radii behavior in silver across *N* = 50 supports the magicity of *N* = 50, the robustness of the nearby *N* = *Z* = 50 shell closure in Sn is still under debate^56–59^.

In conclusion, we performed in-source RIS measurements of the mean-squared charge radii of ^96−104^Ag. The in-source measurements were complemented by high-precision collinear laser spectroscopy of neutron-rich ^114−121^Ag. The in-source measurement required the development of a sensitive laser spectroscopy technique which combines the high mass resolving power of the PI-ICR method with the efficiency of a hot-cavity catcher laser ion source. This work represents a milestone for further studies in the region, namely toward ^94,95^Ag where the properties of the unique isomer of ^94^Ag^(21+)^^7^ at the *N* = *Z* line are still under debate^60^. Furthermore, the charge radii of ^94,95^Ag are required to understand the trend across *N* = 50 and to provide a more stringent test of the different DFT models. The work presented here thus opens a window of opportunity to laser spectroscopic studies of very proton-rich nuclei in the ^100^Sn region.

## Methods

### Laser setup

The three-step laser ionization scheme, presented in Fig. 1, was realized with pulsed titanium:sapphire lasers, constructed in-house, pumped by 10 kHz repetition rate Nd:YAG lasers (Lee Laser LDP 200 MQG) operating at 532 nm. The 328.1624 nm first step transition [Kr]4d^10^5s^2^S_1/2_ → 5p^2^P_3/2_ was used for the laser spectroscopy. Use of a 0.3 mm thick etalon (LaserOptik 40% reflectivity at 950–1050 nm) combined with a 6 mm thick etalon in the titanium:sapphire laser resonator reduced the laser linewidth close to the Doppler distribution in the hot cavity. The 6 mm thick etalon was a polished, uncoated, and undoped YAG crystal with about 10% reflectivity^61^. Both of the etalons were mounted in LiopTec V100R-100-1PCL-BU mounts driven by closed-loop PiezoMike E-472 motors.

The synchronized etalon scanning, required for the broad scan range, was achieved with a lookup table generated manually by optimizing the etalons for maximal output power for a given frequency within the range. A third-harmonic generation setup converted the fundamental laser output to the required wavelength. After the third-harmonic generation, the output power varied from 20 to 5 mW across the scan range due to walk-off losses in the thick etalon. Laser power stabilization was not available during the experiment; however, the scans of ^96,97^Ag used two separate scan ranges with similar average powers in order to maintain the relative intensities of the two resolved peaks.

A standard titanium:sapphire laser utilizing intra-cavity second harmonic generation produced the second resonant step at 421.2142 nm. In order to avoid broadening effects on the 328 nm transition due to heavy second step saturation, the laser power was limited to ~150 mW. A combined output of two titanium:sapphire lasers, operating at ~792 nm fundamental frequency, allowed us to achieve the necessary power to saturate the ionization step. The choice of the wavelength for the ionization step was guided by the highest possible output power, and the possibility to recover population from a state populated by a radiative decay from the second excited state, 6d^2^D_5/2_.

Wavemeters from HighFinesse monitored the laser frequencies. A medium-precision model, WS-6, monitored the titanium:sapphire lasers used for the second and non-resonant ionization step, and a high-precision HighFinesse WSU-10 wavemeter measured the frequency of the spectroscopy step with a nominal accuracy of 10 MHz^62^.

### Ag production with the hot cavity

The hot-cavity catcher features two sections; both operated at around 1800 K during the experiment. First, a catcher assembly consisting of a molybdenum crucible, graphite catcher^63^, and a target stack is heated inductively using an UltraFlex SM-2/200 induction heating system capable of providing up to 2 kW of absorbed heating power. A six turn induction coil wound from a 3 mm copper tube with a ~12 mm diameter opening around the catcher window to allow the passage of the primary beam, delivers the power to the crucible. The coil is separated by ~2 mm from the crucible. Second, a graphite transfer tube, confining the atoms for efficient laser ionization, is heated resistively with ~80 A provided by a Delta Elektronika SM 30–100 D DC power supply. Due to the small cross-sectional area and the resistivity of graphite, the current effectively heats the tube through Joule heating and simultaneously forms a few Volt potential gradient along the tube length^64^. The efficiency of the system was determined to be of the order of 1% after the mass separator, with a few tens of ms mean delay for the release of silver, by implanting a ^107^Ag beam from the K-130 cyclotron^23^.

A ^14^N beam, produced at the Heavy-Ion Ion Source Injector ion source^65^, was accelerated to 148 MeV by the K-130 heavy-ion cyclotron. The beam impinged on a circular target foil stack mounted in the molybdenum crucible of the hot-cavity catcher. The target foil stack, with an effective area of about 64 mm^2^, closed the hot cavity to form a 220 mm^3^ cylindrical enclosure. Depending on the isotope of interest, the stack was formed from either a single 2.6–3.3 mg cm^−2^ ^92/*nat*.^Mo foil or it had a backing foil made from a 3 to 12.5 μm molybdenum. LISE++^66^ simulations guided the choice of the primary beam energy, and the target configuration for different isotopes of interest.

The reaction products recoil from the target and subsequently implant into the catcher. The radioactive silver isotopes promptly diffuse out as atoms into the catcher volume^23^ from where they effuse onward into the graphite transfer tube where a three-step laser ionization scheme selectively ionizes them. The ions are extracted quickly and efficiently into the sextupole ion guide (SPIG)^67^. The ions guided via the SPIG are electrostatically accelerated to 30 keV and transported to the dipole magnet. After the mass separation, the continuous ion beam with a selected mass-to-charge ratio *A*/*q* is converted into a bunched-beam by a gas-filled radiofrequency quadrupole cooler and buncher^68^ before injection into the JYFLTRAP double Penning trap^24^ placed in a 7-T superconducting solenoid.

### PI-ICR-based RIS

RIS experiments have recently begun to utilize multi-reflection time-of-flight devices for isobaric purification to achieve high counting sensitivity^14^^,^^69^. In this work, we developed a technique to harness the high mass resolving power of the PI-ICR^17^ method, recently implemented at JYFLTRAP^70^, for RIS.

The ions injected into the trap (see Fig. 1) were first cooled, centered, and additionally purified from isobaric contaminants in the preparation trap via a mass-selective buffer gas cooling technique^71^ before being transported to the measurement trap where the ion’s mass-dependent cyclotron motion was excited. After a particular phase accumulation time, different mass states in the ions accumulate a phase difference. Releasing the ions at this point projected the radial ion motion in the trap onto a position-sensitive detector (MCP detector with a delay line anode), placed outside the strong magnetic field^70^.

In an ideal projection, the magnified image on the detector represents the ion motion in the trap and preserves the phase angle between the states. Thus, different ion landing regions on the detector correspond to different ion states (as an example, see inset in Fig. 1). The ions of one species, either the ground state or the isomeric state, can be chosen for analysis by applying software gates on ion landing coordinates. Furthermore, correlating the ions to the tagged laser frequency allows the construction of a hyperfine spectrum.

### Analysis procedure for in-source RIS

An in-house written analysis software combined the separate scans exported from the JYFLTRAP data-acquisition program, Pymasscanner. Furthermore, the software binned the data and converted the total counts per bin to an ion rate as a function of the laser frequency using the trap pattern duration, thus resulting in the final experimental hyperfine spectrum.

The SATLAS package^72^ was used to fit a calculated hyperfine spectrum to the experimental data with transition frequencies related to the fine-structure transition frequency given as

2 $${{\Delta }}\nu \,=\,A\,\times\, \frac{C}{2}\,+\,B\,\times\, \frac{\left.3C(C\,+\,1)\,-\,4I(I\,+\,1)J(J\,+\,1)\right)}{8I(2I\,-\,1)J(2J\,-\,1)},$$

where *J* is the atomic spin, *I* is the nuclear spin, *A* and *B* are the hyperfine coupling constants, and *C* = *F*(*F* + 1) − *I*(*I* + 1) − *J*(*J* + 1), with *F* the spin of a hyperfine state. Both the atomic ground and excited state exhibit a hyperfine splitting, yielding a total of six transitions per isotope. However, the limited resolution for the hot-cavity measurements means only two peaks, each composed of three transitions, could be resolved. Regular reference measurements on ^109^Ag in between the measurements of other isotopes provided the Gaussian and Lorentzian components full-width-half-maximum for the Voigt profile as well as the reference centroids *ν*^109^ for the fits. As the upper-state hyperfine structure remained unresolved, setting the ratio of upper and lower hyperfine coefficients^19^ to *A*_*u*_/*A*_*l*_ = 0.0186, allowed further restrictions on the degrees of freedom. Relative intensities of the unresolved peaks were fixed to theoretical values^11^, but the relative intensity of the two distinct peaks remained as a free parameter. Known hyperfine *B* coefficients were used for the ^2^P_3/2_ state where available^30^. For ^102^Ag, the coefficient was interpolated from ^103^Ag and ^101^Ag. For *A* < 101, the value was fixed to 25 MHz, the same as for ^101^Ag. The choice of *B* did not have a large effect on the extracted *A*_*l*_ or *ν*^*A*^. The background was fixed to 0 for the low-statistics cases ^99*m*,96^Ag. The bootstrapping method provided the variance of the fit parameters. For each isotope, the data was resampled with repetitions and Eq. (2) was fitted 5000 times for multiple bin widths. The distribution of the fit parameters obtained for these samples provided the parameter uncertainties, and justified the choice of the bin width. Finally, Eq. (2) was used to extract the charge radii. The values shown in Fig. 2b are the median of the resampled values from the bootstrapping process, and the error bars correspond to the 16th percentile and the 84th percentile.

### Collinear laser spectroscopy

The charge radii of the neutron-rich silver isotopes were measured using bunched-beam collinear laser spectroscopy at the IGISOL collinear laser spectroscopy setup^73,74^. The isotopes of interest, produced by proton-induced fission of natural uranium, recoil from the target and are stopped in a high-pressure (300 mbar) helium buffer gas. These fission fragments are then guided towards the SPIG by the gas flow, accelerated to 30 keV, mass separated using a dipole magnet, and injected into the gas-filled RFQ cooler-buncher. Here, the ions accumulate for 100 ms in a potential formed by RF and static electric fields. Lowering the trapping potential releases the ions in bunches with a temporal width of 10 μs, which then enter into the collinear laser spectroscopy beamline. First, the ions neutralize via charge-exchange processes with hot potassium vapor in a charge-exchange cell. The neutral atom bunches are then overlapped anti-collinearly with a continuous-wave laser beam, produced by frequency doubling the fundamental output from a Matisse DS dye laser using a Matisse WaveTrain. Lasing medium mixed from Rhodamine 640 (1.13 g l^−1^) and 6G (0.5 g l^−1^) delivered the required fundamental wavelength of 656 nm. Applying an acceleration voltage to the charge-exchange cell alters the laser wavelength observed in the rest-frame of the atoms prior to the neutralization process. Recording the number of photons observed by a segmented Photo-Multiplier Tube as a function of this Doppler-shifted laser frequency enables the measurement of the hyperfine spectra. The analysis of the hyperfine spectra follows the description given for the in-source measurements. The higher resolution offered by the collinear method allows it to resolve all the resonances cleanly; thus, all hyperfine parameters and relative peak intensities can be left to vary during the fit. Regular reference measurements, either on ^109^Ag or ^117^Ag, were performed to correct for slow drifts in, e.g., the wavemeter calibration and to obtain reliable isotope shifts. However, no significant drifts were observed in the centroids of the reference spectra, simplifying the extraction of isotope shifts from the data.

### Theoretical calculations

In this work, the DFT calculations for silver isotopes were done in a Hartree-Fock-Bogoliubov framework using the Fayans functional Fy(Δ*r*, HFB)^43^, and two other Skyrme EDF models, UNEDF0^41^, and UNEDF2^42^. The numerical calculations, performed with the computer code HFBTHO^75^, utilizes an axially symmetric harmonic oscillator basis. For the Fy(Δ*r*, HFB) functional, a version of the code adjusted to the Fayans functional, was used. Since all of the isotopes were odd-*A* or odd–odd nuclei, the calculations used quasiparticle blocking with an equal filling approximation^76^.

As a first step, the position HFB energy minimum, as a function of axial deformation, was located for the reference even–even palladium isotopes. Next, a set of quasiparticle blocking configurations were calculated for each odd and odd–odd isotope, from which the one with the lowest energy was picked. Due to the Fy(Δ*r*, HFB) functional being initially adjusted with a coordinate space code, the pairing strength parameters had to be readjusted for a basis-based code, stemming from the fact that the level density in the quasiparticle continuum differs notably in-between these two approaches. All pairing channel EDF parameters were scaled with the same coefficient to reproduce the empirical neutron pairing gap in the mid-shell *N* = 70 Pd isotope.

## Data Availability

The data represented in Fig. 2 are available as Source Data. All other data that support the plots within this paper and other findings of this study are available from the corresponding author upon reasonable request. Source data are provided with this paper.

## Source data

Source Data

## Code availability

All the codes for the experimental data used in this study are available from the authors upon reasonable request.
